# Biomarkers and Utility of the Antioxidant Potential of Probiotic Lactobacilli and Bifidobacteria as Representatives of the Human Gut Microbiota

**DOI:** 10.3390/biomedicines9101340

**Published:** 2021-09-28

**Authors:** Olga V. Averina, Elena U. Poluektova, Mariya V. Marsova, Valery N. Danilenko

**Affiliations:** 1 Vavilov Institute of General Genetics, Russion Academy of Sciences, 119991 Moscow, Russia; epolu@vigg.ru (E.U.P.); masha_marsova@mail.ru (M.V.M.); valerid@vigg.ru (V.N.D.); 2 Institute of Ecology, Peoples’ Friendship University of Russia (RUDN University), 117198 Moscow, Russia

**Keywords:** lactobacilli, bifidobacteria, probiotic, oxidative stress, antioxidant, gut microbiota

## Abstract

Lactobacilli and bifidobacteria are an important part of human gut microbiota. Among numerous benefits, their antioxidant properties are attracting more and more attention. Multiple in vivo and in vitro studies have demonstrated that lactobacilli and bifidobacteria, along with their cellular components, possess excellent antioxidant capacity, which provides a certain degree of protection to the human body against diseases associated with oxidative stress. Recently, lactobacilli and bifidobacteria have begun to be considered as a new source of natural antioxidants. This review summarizes the current state of research on various antioxidant properties of lactobacilli and bifidobacteria. Special emphasis is given to the mechanisms of antioxidant activity of these bacteria in the human gut microbiota, which involve bacterial cell components and metabolites. This review is also dedicated to the genes involved in the antioxidant properties of lactobacilli and bifidobacteria strains as indicators of their antioxidant potential in human gut microbiota. Identification of the antioxidant biomarkers of the gut microbiota is of great importance both for creating diagnostic systems for assessing oxidative stress and for choosing strategies aimed at restoring the normal functioning of the microbiota and, through it, restoring human health. In this review, the practical application of probiotic strains with proven antioxidant properties to prevent oxidative stress is also considered.

## 1. Introduction

Oxidative stress (OS) is a common pathogenetic mechanism of tissue damage, and is one of the main factors affecting the course of many diseases. OS is caused by the main reactive oxygen species (ROS): superoxide radicals, hydroxyl radicals (HO·), lipid peroxide radicals (LOO·), and hydrogen peroxide (H_2_O_2_). Endogenous ROS are a byproduct of metabolism that occurs naturally inside the cell during oxygen metabolism as a part of cellular respiration carried out by the mitochondria—free oxidation (FRO) of biomolecules, including proteins, lipids, and nucleic acids [1,2,3]. Exogenous ROS are the result of many negative external factors: environmental pollution, radiation, drugs, bacterial infection, excessive iron intake, imbalance in the intestinal microbiota, etc. [1,4]. In order to neutralize the pro-oxidants, the human body synthesizes antioxidant (AO) enzymes and molecules that form a natural biological AO barrier. AOs interact with free radicals generated in cells, and prevent the chain reactions caused by active oxygen species from disrupting cell functions. However, when the membranes and mitochondria are damaged, the amount of ROS increases dramatically. OS is defined as a condition in which the pro-oxidant–antioxidant balance in the cell is disturbed, resulting in DNA hydroxylation, protein denaturation, lipid peroxidation, and apoptosis, ultimately compromising cells’ viability. OS is accompanied by various inflammatory processes, and is involved in a large variety of disorders, including many chronic conditions, such as cardiovascular, respiratory, neurological, and inflammatory diseases [1,5].

Oxidative damage is a critical cause of inflammation, as well as age-related diseases. Age-related inflammation is described as a chronic, slow, systemic proinflammatory condition characterized by elevated levels of cytokines and inflammatory mediators. This type of inflammation lies at the heart of a wide range of age-related pathologies, including atherosclerosis, cancer, emphysema, liver cirrhosis, arthritis, neurodegenerative and cardiovascular diseases, chronic obstructive pulmonary disease, chronic kidney disease, etc. [1,5]. Chronic lung diseases, including those provoked by infectious and toxic agents, develop as a result of excessive amounts of ROS formed following chronic activation of the immune system [6]. OS and oxidative modification of proteins are common features of almost all cardiovascular pathologies, including myocardial infarction, stroke, and peripheral vascular diseases [7]. Reperfusion induces tissue reactions that fuel the production of ROS, sequestration of proinflammatory immunocytes in ischemic tissues, endoplasmic reticulum stress, and the development of postischemic capillary “no-reflow”, which exacerbate tissue damage. Thus, OS is one of the most important pathological mechanisms of reperfusion injury, which causes apoptosis, autophagy, inflammation, and other tissue damage in several ways, ultimately causing irreversible damage to organs [8,9,10]. Ischemic/reperfusion injury is the most common cause of disease and death, and is a widespread problem in organ transplantation [11,12].

OS also contributes to and accompanies the development of neurodegenerative diseases. The central nervous system consists of some of the most metabolically active tissues of the body. Consumption of large amounts of oxygen by brain cells inevitably leads to the formation of a large number of its active forms. Nerve tissue is particularly sensitive to OS, which is associated with the peculiar properties of neuronal metabolism, mitochondrial dysfunction, and neuroinflammation caused by the activation of microglia, astrocytes, and other brain cells in response to the presence of various antigens, including microbial agents [13]. OS serves as a trigger for neurodegenerative brain diseases, such as Parkinson’s disease, Alzheimer’s disease, amyotrophic lateral sclerosis, and others [14]. In addition, neurodegenerative disorders are associated with the intestinal microbiota, which engage in a bidirectional communication as part of the gut–brain axis [15,16]. OS is also a major cause of depression [17,18]. Some studies suggest that depression is the clinical expression of activated immune-inflammatory, oxidative, and nitrosative stress pathways, including tryptophan catabolites, accompanying autoimmune reactions and increased bacterial translocation [19]. One of the possible biochemical mechanisms underlying the development of depression is the deregulation of the kynurenine pathway as a result of an increase in proinflammatory cytokine levels and the development of OS. These factors lead to a deficiency of serotonin and melatonin, which is considered to be one of the main causes of depression [20]. Increased inflammation and OS have been identified repeatedly in depression [21,22,23].

Considering the central role played by OS in many diseases, there is an urgent need to find novel solutions. The use of probiotics is being considered for the reduction of OS in the human body. Lactobacilli and bifidobacteria constitute an important probiotic component of the gut microbiota. They improve epithelial and mucosal barrier functions, regulate the composition of intestinal microbiota, and inhibit excessive proliferation of harmful bacteria [24]. Multiple in vivo and in vitro studies have demonstrated that lactobacilli and bifidobacteria possess excellent AO capacity, providing a certain degree of protection against oxidative stress [25,26]. Recently, lactobacilli and bifidobacteria have been considered as a new source of natural AOs. The ability of probiotic bacteria to produce AO enzymes and metabolites makes them the most promising of all means against the free radicals. New perspectives emerge when lactobacilli and bifidobacteria are used not in the form of live cultures of probiotic bacteria, but in the form of postbiotics—metabolites and cell components with antioxidant activity [27].

Lactobacilli and bifidobacteria, as regular inhabitants of the gastrointestinal tract (GIT) in both humans and animals, can exert AO activity via various mechanisms: chelation of toxic ions (Fe^2+^ and Cu^2+^); synthesis of AO enzymes, peptides, and thiols; compounds with AO properties; effects on cell receptors and regulation of internal signal transduction systems of eukaryotic cells; activation of transcription of enzymes that neutralize free radicals; modulation of species composition of the gut microbiota; and impact on the permeability of the intestinal barrier [28,29].

Many recently published reviews describe the AO properties of lactobacilli and bifidobacteria, along with the mechanisms and signaling pathways employed by them to prevent oxidative damage. Moreover, the genes associated with lactobacilli and bifidobacteria redox potential, as well assuitable methods to evaluate bacteria’s AO capacity, are being actively discussed [29,30,31]. This review considers the biomarkers of the AO potential of human gut probiotic lactobacilli and bifidobacteria, and their practical application, for the prevention of OS. Its main goal is to summarize the current state of the research on AO compounds derived from lactobacilli and bifidobacteria. Special emphasis is given to the mechanisms of AO activity used by probiotic lactobacilli and bifidobacteria, as well as their metabolites in the human gut. The AO properties of lactobacilli, bifidobacteria, and their components have been shown in multiple in vivo and in vitro studies. This review scrutinizes the known functional AO enzymes, metabolites, and cellular compounds of lactobacilli and bifidobacteria strains for a better understanding of their role in protecting the host and its own cells from OS. The genes encoding these AOs can be selected and used for carrying out metagenomic analysis of the gut microbiota, and to identify biomarkers of the AO potential of probiotic bacteria in various diseases accompanied by OS. The identification of AO biomarkers of probiotic bacteria is of great importance both for creating diagnostic systems for identifying OS, and for choosing strategies aimed at restoring normal functioning of the microbiota and restoring human health.

## 2. Lactobacilli and Bifidobacteria as Members of the Human Gut Microbiota

The gut microbiota plays a vital role in human health and pathology, and accordingly is a popular area of scientific research [32]. Bifidobacteria [33,34] and lactobacilli [35] are the most important probiotic bacteria of the gut microbiota. Their positive functions include antagonism and competition to opportunistic pathogens, improving digestion, participation in maturation of the immune system in early life and preservation of immune homeostasis during life, neuromodulation, and production of vitamins and other beneficial compounds, including AOs [36,37]. These bacteria can exhibit substantial AO activity in the host intestine, and promote the production of AO enzymes and compounds that act by neutralizing ROS and prevent oxidative damage [6,12]. However, most of their functions are strain-specific and not common to multiple genera or species [6,12,13].

The genus *Bifidobacterium* is one of the predominant bacterial populations in human gut microbiota. The abundance of bifidobacteria in vaginally delivered breast-fed infants amounts to 90% of the total gut microbiota. During life, the bifidobacterial count in the colon of adults drops to 5%, and decreases even further in elderly people [36]. Many studies show that the abundance of bifidobacteria is lower in the gut microbiota of patients with various disorders, such as inflammatory bowel diseases, autism spectrum disorders (ASD), depression, and others [38,39].

Bifidobacteria play a major role in maintaining a healthy human gut microbiome [36,40]. One important function of the bifidobacterial genus is the production of acetate and lactate after carbohydrate fermentation, which can be converted into butyrate by other colonic bacteria through cross-feeding [41].

The species *Bifidobacterium adolescentis*, *Bifidobacterium longum* subsp. *longum* (*B. longum*), *Bifidobacterium longum* subsp. *infantis* (*B. infantis*), *Bifidobacterium pseudolongum*, *Bifidobacterium catenulatum*, *Bifidobacterium pseudocatenulatum*, *Bifidobacterium breve*, *Bifidobacterium bifidum*, *Bifidobacterium animalis* subsp. *lactis* (*B. lactis*), *Bifidobacterium dentium*, and *Bifidobacterium angulatum* are isolated from stool samples of healthy humans [40]. Bifidobacteria are anaerobes that colonize anoxic environments such as that of the colon, but their sensitivity to oxygen has been shown to vary between different species. For instance, *B. lactis* is considered oxygen-tolerant, while *B. bifidum*, *B. breve*, and *B. longum* are oxygen-sensitive (grow in the presence of 5% O_2_ in liquid culture), and *B. infantis* and *B. adolescentis* are oxygen-hypersensitive (growth inhibited in 5% O_2_ conditions) [42].

Lactobacilli are Gram-positive microorganisms incapable of spore formation. They are isolated from plants and plant products, silage, dairy products and milk, fermented food products (cheese, olives, pickles, salami, etc.), the oral cavity, and the vagina and the gut of humans and animals, as well as from household and industrial waste. Lactobacilli are divided into free-living, host-adapted, and “nomadic” species. Nomadic species are not autochthonous in the classical sense, but they are adapted to the ecosystems of the GIT and oral cavity, which allows them to survive there for a long time [43]. In adult feces, lactobacilli account only for 0.01–0.06% (10^5^ to 10^8^ CFU/g) of all bacterial species. Despite constituting an insignificant part of the gut microbiota, lactobacilli are a permanent and essential component. The predominant indigenous *Lactobacillus* species are *Lactobacillus* *gasseri*, *Limosilactobacillus reuteri*, *Lactobacillus crispatus*, *Ligilactobacillus salivarius*, and *Ligilactobacillus* *ruminis*. Gut-dwelling lactobacilli encompass *Lactocaseibacillus* *casei*, *Limosilactobacillus plantarum*, *Limosilactobacillus fermentum*, and *Lactocaseibacillus rhamnosus*. The most common isolates from the stomach mucosa are *Limosilactobacillus* *antri*, *Limosilactobacillus gastricus*, *Lactobacillus kalixensis*, *L. reuteri*, and *Lactobacillus* *ultunensis*. The species *L. crispatus*, *L. gasseri*, *Lactobacillus jensenii*, *Limosilactobacillus vaginalis*, and *Lactobacillus iners* are frequently found in the vagina [44]. A thorough study, using whole-genome sequencing, identified 86 *Lactobacillus* strains belonging to 52 species in human feces; 43 of those species occupied the GIT as permanent residents [45].

Lactobacilli are facultative anaerobes or microaerophiles. It has been demonstrated that some strains are able to use oxygen as a substrate in reactions mediated by flavin oxidases and, in some cases, to synthesize a minimal respiratory chain. The occurrence of genes related to aerobic (oxygen) and respiratory (oxygen, exogenous heme, and menaquinone to activate a minimal electron transport chain) metabolism correlates with the taxonomic classification of lactobacilli. Aerobic and respiratory metabolism was reported in *L. casei*, *L. plantarum*, *Lactobacillus johnsonii*, *Lentilactobacillus buchneri*, and *L. reuteri*. The shift from anaerobic growth to aerobic and/or respiratory offers physiological advantages, and affects the metabolite profiles of several species. Despite the fact that these differences do not necessarily result in increases in biomass production or growth rate, the cells grown in these conditions exhibit frequently improved tolerance to heat and OS [46].

OS contributes significantly to dysbiosis by reducing the microbial diversity of the gut microbiota [47,48]. The gut microbiota can regulate redox signaling and redox homeostasis in the host [49]. Lactobacilli and bifidobacteria, as regular inhabitants of the GIT of both humans and animals, can regulate the composition of the intestinal microbiota and inhibit excessive proliferation of harmful bacteria, which may contribute to decreasing OS.

Lactobacilli and bifidobacteria can reduce the intestinal pH and suppress the growth of various pathogenic bacteria to maintain the balance of the gut microbiota [50]. Additionally, some strains of lactobacilli and bifidobacteria produce a variety of substances toxic to pathogenic microorganisms, such as organic acids, bacteriocins, and biosurfactants [51]. Lactobacilli and bifidobacteria compete with pathogens for nutrients and adhesion to intestinal epithelial cells. The collagen surface-binding protein produced by *L. fermentum* RC-14 inhibits the adhesion of *Enterococcus faecalis* 1131 to epithelial cells [52]. Dietary alteration of the gut microbiota is strongly linked to OS. Mice fed a high-fat diet and treated with lipoic acid displayed decreased ROS levels and increased total AO capacity, which were positively associated with lactobacilli and negatively correlated with *Escherichia coli* and enterococci. Supplementation of *L. johnsonii* BS15 alleviated high-fat diet-induced OS and altered the intestinal *Firmicutes/Bacteroidetes* ratio in mice, suggesting that modulation of the gut microbiota by lactobacilli has the potential to improve the host redox state [30]. Probiotic lactobacilli and bifidobacteria are able to change the composition of the intestinal microbiota, tipping the balance towards a higher abundance of useful bacteria. A probiotic formulation comprising *L. rhamnosus* GG, *L. acidophilus*, *L. plantarum*, *L. paracasei*, and *Lactobacillus delbrueckii* increased the content of bacteria such as *Prevotella* and *Oscillibacter*, displaying anti-inflammatory activity in the gut microbiota of rats [53]. The consumption of *B. longum* BB536 altered the gut luminal biotin content and butyrate metabolism by changing the gut microbiota composition [54]. Wang et al. examined the AO activity of the strain *B. bifidum* ATCC 29521—a species typical of the colonic microflora of humans—in the intestinal tracts of mice, by evaluating changes in the gut microbiota composition and ROS levels in their intestinal contents for 28 days of oral administration. *B. bifidum* ATCC 29521 significantly (*p* < 0.05) improved the ecosystem of the intestinal tract of BALB/c mice by increasing the amount of probiotic bacteria and by reducing unwanted bacterial populations [55].

## 3. In Vitro and In Vivo Study of Antioxidant Properties of Lactobacilli and Bifidobacteria

The AO properties of intact cells of lactobacilli and bifidobacteria, as well as their cell-free extracts (supernatants), intracellular extracts, metabolites, and cell wall components, have been demonstrated in both in vitro and in vivo studies (Table 1).

The main assays commonly used in in vitro studies are DPPH radical scavenging, the cellular antioxidant activity (CAA) assay, inhibition of linoleic acid peroxidation (ILAP), hydroxyl radical scavenging (HRS), and reducing power (RP) assays, among others. In the study of Amaretti et al. [28], the strains *Levilactobacillus brevis*, *L. acidophilus*, and *B. lactis* were selected as those displaying the highest levels of AO activity. Oxygen radical absorbance capacity (ORAC) was found to be a highly strain-specific feature characteristic of *B. longum* CUETM 172 [56]. Another study [57] investigated the AO effects of intracellular extracts and intact cells of *L. acidophilus* ATCC 4356 and *B. longum* ATCC 15708. Both experiments attested to a strong AO capacity of these strains. Proteins isolated from *Bifidobacterium* *animalis* subsp. *animalis* 01 were shown to possess AO activity [58]. The AO potential of supernatants, intact cells, and the intracellular extract of *B. animalis* 01 was also evaluated in the study of Chen et al. [59]. Lin and Yen [60] revealed in their studies the inhibitory effect of *B. longum* strains on lipid peroxidation. The AO action of cell-free culture supernatants of different *Lactobacillus* strains was evaluated using a test system based on *Escherichia coli* MG1655 strains carrying plasmids encoding luminescent proteins. The majority of strains (51 out of 81) belonging to six different species demonstrated high levels of AO activity [61].

The ability of probiotic bacteria and their metabolites to inhibit the increase in intracellular ROS has been shown in cellular models [62,63]. Some probiotic bacteria can upregulate the intracellular activity of superoxide dismutase (SOD), catalase (CAT), and glutathione peroxidase (GSH-PX) at both the enzymatic and transcriptional levels, and protect the cells from oxidative damage [64]. Pre-incubation with supernatants of *B. longum* CCFM752, *L. plantarum* CCFM1149, or *L. plantarum* CCFM10 significantly suppressed the angiotensin-II-induced increase in ROS levels and increased CAT activity in A7R5 cells, whereas CCFM752 inhibited NADPH oxidase activation, and CCFM1149 enhanced the intracellular SOD activity simultaneously. The supernatant of CCFM752 down regulated the expression of NADPH oxidase activator 1 (Noxa1) and angiotensinogen in A7R5 cells [65]. A study by Choi et al. on HT-29 cells revealed that both polysaccharides and heat-killed *L. acidophilus* 606 demonstrated high AO activities [66].

The antioxidative properties of *Lactobacillus* and *Bifidobacterium* strains have been demonstrated in different animal models. *L. plantarum* KSFY02 and *B. animalis* RH demonstrated AO properties in aged mice. Both strains were found to increase the activities of SOD, CAT, and GSH-PX in this model [67]. The strain *L. brevis* MG000874 was tested on a murine model of D-galactose-induced OS. Animals fed with probiotic bacteria had an increased amount of AO enzymes in all tissues, including glutathione-S-transferase in the liver and blood [68]. In a study by Wanchao using a model of ischemia and reperfusion shock of the brain in Sprague Dawley rats, it was shown that the administration of an inactivated culture of a *Lactobacillus* strain significantly improved neurological parameters, reduced the size of the affected area, reduced the amount of malondialdehyde (MDA), and increased the activity of SOD [69]. Tang et al. investigated the effects of the *L. reuteri* DSM 17938 on the development of OS in a model of necrotic enterocolitis in newborn mice. *L. reuteri* DSM 17938 reduced pathological parameters such as the expression of TNF-α and IL-1β, MDA, GSSG, and the GSSG/GSH ratio, and significantly increased SOD activity and GSH levels in mice [70]. The introduction of *L. brevis* 47f strain to BALB/c mice subjected to 5-fluorouracil (5-FU)-induced mucositis protected enterocytes and decreased MDA blood plasma levels and MDA levels in the intestinal tissues (2–3 times lower than in the positive control group) [71]. The positive AO impact of *L. fermentum* U-21 strain on paraquat-treated C57/BL6 mice was observed in the study of Marsova et al. [72].

Grompone et al. carried out an interesting experiment, the purpose of which was to assess the AO potential of probiotic bacteria in *Caenorhabditis elegans* [73]. In total, 78 strains of *Bifidobacterium* and *Lactobacillus* were examined in the study. One of the most efficient strains was *L. rhamnosus* CNCM I-3690, which protected *C. elegans* from H_2_O_2_-induced OS, increasing their viability by 30% and increasing the average lifespan of worms by 20% [73]. The impact of 86 strains of *Lactobacillus* on the survival rate of *C. elegans* exposed to the oxidant paraquat was studied by Marsova et al. The screening yielded several promising strains possessing high AO activity [61].

The effects of intake of probiotic strains on the reduction in OS and the improvement of AO biomarkers has been investigated in interventional studies [74,75]. A meta-analysis conducted by Heshmati at al. studied the effects of consumption of probiotics and synbiotics on indicators of OS in healthy subjects. The authors concluded that these supplements improve AO resistance and increase the amount of AO enzymes in the human body [76]. Another meta-analysis conducted on patients with chronic kidney disease indicated that bacterial therapy has significant beneficial effects on serum levels of C-reactive protein (CRP), total GSH, MDA, and total AO capacity [77]. A randomized clinical trial conducted by Chamari et al. investigated the impact of probiotics on the CAT plasma levels of healthy women [78]. The probiotic group demonstrated a significant increase in CAT activity in comparison with the untreated control group. The influence of intake of a probiotic containing the strains *B. longum* CECT 7347, *L. casei* CECT 9104, and *L. rhamnosus* CECT 8361 on OS induced by physical exercise (high intensity and duration) was studied in healthy subjects (male cyclists) [79]. Probiotic supplementation contributed to increased plasma AO levels and ROS neutralization.

Many studies have proven that strains of lactobacilli and bifidobacteria, along with their metabolites and cellular components, are capable of reducing OS in the host cells. While the AO activity of probiotic bacteria is not completely understood, some mechanisms have been described in published papers and reviews.

**Table 1:** Examples of the study of AO activity of lactobacilli and bifidobacteria.

Strain and Species of Bacteria	The Investigated Component of Bacteria	Experiment Duration	Cell Lines	Animal Model	Studed Group of People	Tests Used	Research Results	References
*B. animalis 01*	Intact cells, culture supernatant, intracellular cell-free extracts					Inhibition of linoleic acid peroxidation. Scavenge DPPH; scavenging effect on hydroxyl radicals and superoxide anions.	All investigated probiotic forms had AO activity.	[59]
81 Lactobacilli strains of 6 different species	Cell-free culture supernatant					Test system based on *E. coli* MG1655 strains carrying plasmids encoding luminescent biosensors pSoxS-lux and pKatG-lux.	51 strains demonstrated AO activity.	[61]
*B. longum* CCFM752, *L. plantarum* CCFM1149, *L. plantarum* CCFM10	Cell-free culture supernatant		A7R5			Determination of the angiotensin-II-induced ROS levels, catalase NADPH oxidase, and intracellular superoxide dismutase (SOD) activity. Regulation of the expression of NADPH oxidase activator 1 (Noxa1) and angiotensinogen.	Suppression of the angiotensin-II-induced increases in ROS levels (all three strains); Inhibition of NADPH oxidase activation (*B. longum* CCFM752, *L. plantarum* CCFM1149); Enhancement of the intracellular SOD activity (*L. plantarum* CCFM1149); Downregulation of the expression of NADPH oxidase activator 1 (Noxa1) and angiotensinogen (*B. longum* CCFM752).	[65]
*L. acidophilus* ATCC 43121, *L. acidophilus* ATCC 4356, *L. acidophilus* 606, *L. brevis* ATCC 8287, *L. casei* YIT 9029, *L. casei* ATCC 393, *L. rhamnosus* GG	Heat-killed cell (HK); the soluble polysaccharides (SP) components of bacterial cells		Cancer cell lines HT-29, HeLa, MCF-7, U-87, HepG-2, U2Os, PANC-1, hEF			Antiproliferative effects on the cancer cells. Induction of apoptosis. Scavenging activity of the DPPH free radicals.	HK of *L. acidophilus* 606 and *L. casei* ATCC 393 exhibited the most profound inhibitory activity in the all of tested cell lines; SP of *L. acidophilus* 606 evidenced the effective anticancer activity.	[66]
*L. brevis* MG000874	Intact cells, intracellular cell-free extract	8 weeks		Albino mice exposed to D-galactose- induced OS		AO enzymes were quantified in liver, kidney, and serum of animals.	The treated animals displayed improvement in SOD, CAT, and GST in all tissues, as well as GSH in the liver and serum.	[68]
*L. fermentum* U-21	Intact cells	*C. elegans* (1–2 days); mice (23 days)		C57/BL6 mice, *C. elegans* exposed to paraquat-induced OS		The impact on the life span of *C. elegans*; A murine model of Parkinson’s disease	The lifespan of the *C. elegans* was extended by 25%. *L. fermentum* U-21 ensured normal coordination of movements and the safety of dopaminergic neurons in the brain.	[72]
*L. plantarum* A7 (KC 355240, LA7)	Probiotic soy milk, 200 mL/day	8 weeks			24 type 2 diabetic kidney disease patients	Malondialdehyde, 8-iso-prostaglandin F2a, oxidized glutathione, total antioxidant capacity (TAC), reduced glutathione (GSH), glutathione peroxidase, and glutathione reductase were measured in the serum.	Oxidized glutathione concentration was significantly reduced; the levels of GSH, glutathione peroxidase, and glutathione reductase were significantly increased; no significant reduction in the 8-iso-prostaglandin F2α, malondialdehyde and no induction of TAC were detected.	[75]
Various probiotics and synbiotics					27 articles that included 1363 subjects (709 cases and 699 controls)	Total antioxidant capacity (TAC), glutathione (GSH), superoxide dismutase (SOD), nitric oxide (NO), andmalondialdehyde (MDA) were taken into account.	TAC, GSH, SOD, and NO were higher in probiotics (or synbiotics) group compared to controls. MDA level was lower than controls.	[76]
*L. acidophilus*, *L. casei*, *B. bifidum*	Capsules with intact cells (Tak Gen Zist Pharmaceutical Company, Tehran, Iran)	12 weeks			Diabetic hemodialysis patients, 28 cases and 27placebos.	Plasma glucose, serum insulin, assessment-estimated insulin resistance, assessment-estimated beta-cell function and HbA1c, insulin sensitivity, serum C-reactive protein, plasma malondialdehyde, total iron-binding capacity, and plasma total antioxidant capacity were determined.	Patients who received probiotic supplements showed significantly decreased plasma glucose, serum insulin, assessment-estimated insulin resistance and beta-cell function and HbA1c, insulin sensitivity, serum C-reactive protein, plasma malondialdehyde, and total iron-binding capacity. Patients showed an increase in plasma total antioxidant capacity.	[74]

## 4. Mechanisms of Antioxidant Activity of Lactobacilli and Bifidobacteria as the Basis of the Antioxidant Biomarkers

**Figure 1:**
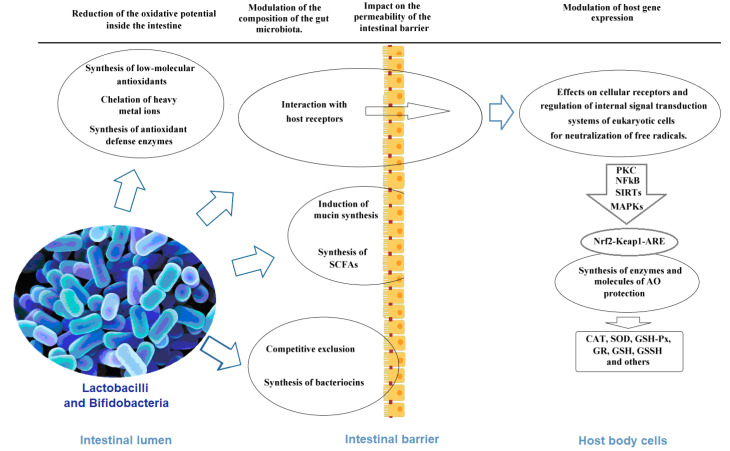
Mechanisms of antioxidant action of lactobacilli and bifidobacteria inside the intestine of the host.AO: antioxidant; OS: oxidative stress; SCFAs: short-chain fatty acids; NF-κB: nuclear factor kappa B, eukaryotic transcription factor; SIRTs: a family of NAD-dependent protein deacetylases; PKC: protein kinase C; MAPKs: mitogen-activated protein kinases; SOD: superoxide dismutase; CAT: catalase; GSH: reduced glutathione; GSSH: oxidized glutathione; GSH-Px: glutathione peroxidase; GR: glutathione reductase; NRF2-Keap-ARE: a signaling system responsible for the expression of antioxidant-responsive elements (ARE-genes).

Recent findings suggest that the main mechanisms employed by probiotic bacteria to reduce OS in their own cells as well as the hosts’ include scavenging ROS, chelation of metal ions, boosting the levels of AO enzymes, synthesis of non-enzymatic AO and metabolites with AO properties, effects on cellular receptors, and regulation of internal signal transduction systems of eukaryotic cells, improving gut permeability. These mechanisms determine the antioxidant potential of probiotic bacteria, and should be used for the selection of genetic biomarkers (Figure 1).

### 4.1. Chelation of Pro-Oxidative Metal Ions

Metal ions are capable of initiating the decomposition of hydrogen peroxide into peroxyl and alkoxy radicals and triggering lipid peroxidation [80]. The chelating capacity of probiotic strains may be explained by the content of certain chelators that are normally detected in the intracellular cell-free extract of bacterial cells [29]. For instance, *L. rhamnosus* GG and *L. paracasei* Fn032 significantly inhibit the production of H_2_O_2_ induced by ferrous ions. *L. casei* KCTC 3260 also possesses a high AO ability via chelating Fe^2+^ or Cu^2+^ [81].

DNA-binding ferritin-like protein (Dps), found in the strain *B. longum* NCC2705, contains conserved iron-binding sites that can carry out chelation of metal ions [82]. Dps proteins are widespread among lactobacilli [83]. One gene encoding a ferroxidase, which was shown to participate in iron chelation, was found in the genome of *B. longum* NCC2705 [84]. The HprA1 protein derived from *L. casei* confers resistance to H_2_O_2_; it binds to Fe^2+^ and prevents the formation of hydroxyl radicals [85]. The *cop*B (export ATPase for copper ions) and *cop*R (inductor-transcription factor) genes are also involved in the response to OS triggered by H_2_O_2_ in *L. plantarum*. It is assumed that these proteins are associated with the chelation of copper ions [86]. The chelating activity of lactobacilli and bifidobacteria is strain-specific [87].

### 4.2. Synthesis of Antioxidant Enzymes

AO enzymes, as a major part of the AO defense system, are deficient in obligate anaerobic bacteria such as bifidobacteria and lactobacilli.

Superoxide dismutase (SOD) is the most efficient enzyme capable of inactivating the superoxide anion. It decomposes the superoxide anion into molecular O_2_ and hydrogen peroxide (H_2_O_2_) using Zn/Cu, Fe/Mn, and Ni as cofactors. MnSODs have been found in several species of lactobacilli, whereas FeSODs and Cu/ZnSODs are not that common. It has been demonstrated that the activity of MnSODs is dependent on the intracellular concentration of Mn^2+^ [66]. Only a few species of lactobacilli—such as *L. casei*, *L. paraplantarum*, *L. buchneri*, *L. sakei*, and *L. brevis*—exhibit SOD activity. Interestingly, SOD activity was found to be absent from the strain *L. casei* Shirota [88]. The selection of promoters and multi-copy *sod*-expressing cassettes allowed the gene *sod* to be cloned from *L. casei* LC2W into *S. thermophilus*. The activity of the SOD enzyme was increased in the genetically modified strain, which was more resistant to H_2_O_2_ [89], proving that the *sod* gene of *L. casei* is active and plays a role in resistance to H_2_O_2_.

Catalase (CAT) is the enzyme that decomposes H_2_O_2_ into water and oxygen. CAT also oxidizes low-molecular alcohols and nitrites in the presence of H_2_O_2_. Although the majority of lactobacilli are CAT-negative, genes coding for catalases have been detected in a number of lactobacilli. CAT in lactobacilli contains a heme group or Mn (less frequently) as a prosthetic group [46,90]. An in silico analysis of 321 genomes of industrially relevant lactobacilli demonstrated that genes of heme-catalase were widespread among the *L. brevis*, *L*. *plantarum*, and *L. sakei* groups, while the Mn-catalase was present only in several strains such as *L. casei* and *L. zeae* [91]. *L. casei* N87 contains two CAT genes—heme- and Mn-type—which are rare in lactobacilli [92]. *L. brevis* has been described as CAT-negative regardless of the fact that an endogenous heme-dependent CAT has been identified by heterologous expression in *L. casei*. Indeed, *L. brevis* cannot synthesize heme due to lack of the enzymes for protoporphyrin IX synthesis, and therefore loses its CAT activity. *L. brevis* catalase can be activated by exogenous gemin. Activated CAT provides protection against the toxicity of H_2_O_2_ [93]. It has been demonstrated that the activity of heme-CAT depends on concentration of hematin [94]. In lactobacilli, the evolution of heme-containing proteins is still poorly understood, and further studies are needed in order to determine whether the genes encoding heme-catalases, such as those encoding the cytochrome oxidase, have been horizontally acquired from aerobic donors, or whether they endured gene loss events [92].

So far, no genes encoding CAT and SOD have been annotated in databases of genome sequences of bifidobacteria [95].

The NADH oxidase/NADH peroxidase system (NOX/NPR) also prevents oxygen accumulation in bacterial cells by producing H_2_O_2_ via NOX and then reducing it to water via NPR. These O_2_-consuming enzymes are responsible for the rapid elimination of O_2_, and play an important role in maintaining the intracellular redox balance. The activity of the NOX/NPR system contributes to the maintenance of the NADH/NAD+ balance by promoting cofactor regeneration. The occurrence of *nox* genes is limited among lactobacilli. H_2_O-forming NOX is widespread in the *L. casei* group (a single sequence is generally present), while some *L. plantarum* strains harbor multiple genes for the NOX (*nox*1, *nox*2, *nox*3, *nox*4, *nox*5, *nox*6). In *L. plantarum* WCFS1, NOX activity during aerobic growth is exclusively determined by the *nox*5 gene [96,97,98]. All *L. plantarum* strains harbor two NPRs (*npr*1, *npr*2), but some studies have revealed that only *npr*2 (lp_2544) is upregulated during aerobic growth [98]. In *L. casei* IGM394, the mechanism of H_2_O_2_ tolerance is solely dependent on NPR [96].

NOX and the oxygen-dependent coproporphyrinogen III oxidase are involved in the detoxification of molecular oxygen and/or H_2_O_2_ in *B. animalis* [99,100]. Other bifidobacteria display reduced NOX and NPR activities [101].

Thioredoxins (Trxs) are reductases that catalyze protein disulfide/dithiol conversion with a conserved -CGPC- active site motif. The Trxs and GSH-glutaredoxin systems play important roles in defense against OS by maintaining intracellular dithiol/disulfide homeostasis in both prokaryotic and eukaryotic cells. They are involved in transfer of electrons to thiol-dependent peroxidases, thereby maintaining redox homeostasis. The Trx system includes thioredoxin reductase, NADPH, thioredoxins, and thioredoxin peroxidase. The thioredoxin-dependent reduction system reduces a number of proteins, including peroxiredoxins, by directly reducing H_2_O_2_, scavenging hydroxyl radicals, quenching singlet oxygen, and maintaining the intracellular thiol–disulfide balance [102]. The thioredoxin system plays an essential role in DNA and protein repair by reducing ribonucleotide reductase and methionine sulfoxide reductases, and regulates the activity of numerous redox-sensitive transcription factors [103].

Peroxiredoxins are enzymes containing a redox-active cysteine site that are oxidized by the peroxide substrate. The recycling back to thiols is reduced by thioredoxin. In bacteria, peroxiredoxins are frequently referred to as alkyl hydroperoxide reductase (AhpC); other names are also used, such as thiol-specific antioxidant (TSA) or thioredoxin peroxidase (TPx). Many bacteria also express the flavoprotein AhpF, which acts as a disulfide reductase that recycles bacterial peroxiredoxins. Peroxiredoxins constitute an important component of the bacterial defense system against toxic peroxides. AhpC is localized in the cytoplasm, and has a wide substrate range that includes H_2_O_2_, organic peroxides, and peroxynitrite. This enzyme participates in the control of endogenous peroxides, as well as in the inducible defense response to exogenous peroxides or general stresses.

Transcriptomic analysis indicates that peroxiredoxins and thioredoxin reductase are potent defense systems against H_2_O_2_-induced stress in *B. longum* and *B. lactis* species [104]. This ability is determined by a gene encoding the AhpC subunit of alkyl hydroperoxide reductase, which reduces the content of H_2_O_2_ and protects cells from OS [105]. Alkyl hydroperoxide reductase is the main scavenger of endogenous H_2_O_2_ generated during aerobic cultivation of *B. longum* [106,107,108]. Sequencing of the genome of *B. longum* revealed the presence of the *trl* gene encoding thioredoxin reductase (NADPH) which, together with alkyl hydroperoxide reductase, is supposedly involved in the elimination of H_2_O_2_ and reduction of glutaredoxin [75,77]. The genomes of the strains *B. longum* LTBL16, *B. longum* NCC2705, and *B. lactis* were found to contain a gene encoding peroxiredoxin Q/BCP, which eliminates ROS [104,107,109]. The thioredoxin-dependent AO system might be the principal redox homeostasis system in the strain *B. longum* BBMN68, in which the gene encoding a thioredoxin reductase was highly upregulated after 60 min of exposure to oxygen [105].

The thioredoxin–thioredoxin reductase system is the major thiol/disulfide redox system in *Lactobacillus* strains, and genes encoding thioredoxin (TRX) and thioredoxin reductase (TR) are present in almost all sequenced strains. Global transcriptomic analyses revealed that, following exposure of *L. plantarum* CAUH2 to H_2_O_2_-induced stress, the expression of thioredoxin reductase increased 36.76-fold. Some transcriptional regulators (Spx, CcpA, and MarR1) were also predicted to be involved in the adaptive response to H_2_O_2_ [110]. In *L. plantarum* WCFS1, overexpression of the thioredoxin reductase gene (*trx*B1) resulted in a higher TR activity and an increased resistance to OS, which was accompanied by induced transcription of 16 genes associated with OS response. The authors suggested that thioredoxin reductase is a pivotal enzyme in the OS response in *L. plantarum* WCFS1 [111]. Normally, the genome of lactobacilli contains several thioredoxin genes, and their functions differ from one another. The *L. casei* Shirota strain possesses four putative thioredoxin genes and one putative thioredoxin reductase gene. Mutants in different *trx* genes show different properties and different reactions to the induction by H_2_O_2_ OS [112]. The *trx* and *trx*R genes may be localized on plasmids of lactobacilli [113]. The genomes of lactobacilli contain the peroxiredoxin gene (*ahpC*) [114]; they also contain a *dsb*A gene encoding a bacterial thiol disulfide oxidoreductase. DsbA catalyzes intrachain disulfide bond formation as peptides emerge into the cell’s periplasm [113].

*B. bifidum*, which grows well under aerobic conditions, contains a homologue of b-type dihydroorotate dehydrogenase (DHOD) composed of PyrK (31 kDa) and PyrDb (34 kDa) subunits. The purified enzyme catalyzed a H_2_O_2_-forming NADH oxidase reaction in the presence of O_2_. It was suggested that the enzyme could be involved in H_2_O_2_ production in bifidobacteria in highly aerated environments [114].

Possible class I pyridine nucleotide disulfide oxidoreductase (PNDR) is involved in the cellular response to OS detected in the *B. longum* NCC 2705 and *B. longum* BBMN68 strains [105,115]. The genes encoding peroxide oxidoreductase (LTBL16-000027, LTBL16-000028, and LTBL16-000976) and NADH oxidase (LTBL-001911) were identified in the genome of the *B. longum* LTBL16 strain, which can effectively eliminate ROS in bifidobacteria and increase resistance to oxygen [116].

The mentioned basic AO enzymes are widespread among lactobacilli and bifidobacteria, but other enzymes also exist. Unfortunately, many of these enzymes remain unidentified. Apart from AO enzymes, probiotic bacteria can produce non-enzymatic AOs.

### 4.3. Non-Enzymatic Antioxidants

Thiol compounds containing two cysteine molecules play an important role in AO protection conferred by lactobacilli and bifidobacteria; these are compounds of the glutathione and thioredoxin groups. Glutathione (GSH) is a tripeptide of gamma-glutamyl-cysteinyl-glycine, synthesized from amino acids. GSH is involved in maintaining the cellular redox status [117,118]. Genetic analysis shows that the first of the GSH synthesis genes—the gamma-glutamylcysteine synthetase gene (glutamate–cysteine ligase, *gsh*A)—is found in many lactobacilli, while the second gene—alpha-L-glutamate ligase (*gsh*B)—is absent. A number of bacteria—especially *S. thermophilus*—have a multidomain bifunctional fusion glutamate–cysteine ligase/glutathione synthetase gene (*gsh*AB, *gsh*F) encoding a protein of ~700 AA, capable of performing both reactions. Lactobacilli also possess a similar protein (for example, GshAB of *L. plantarum* subsp. *plantarum* P-8, 751 AA); however, despite similarity in size and partial homology with *Streptococcus* proteins, it exhibits only glutamate–cysteine ligase function. These data indicate that lactobacilli are not capable of synthetizing GSH de novo. Whole genome sequences of 26 food-grade lactic acid bacteria (LAB) confirmed that all of the strains tested were incapable of GSH synthesis, but could import it from their environment [119]. However, when the *gsf*F genes from *L. plantarum* and *L. casei* were cloned and expressed in *E. coli* cells, GSH titers were enhanced significantly, demonstrating that putative GshF from *Lactobacillus* exert functional activity on GSH biosynthesis [120]. Lactobacilli import GSH from the growth medium by using the CysCD ABC transporter ATP-binding proteins [121]. Despite the inability to synthesize GSH, lactobacilli possess enzymes of glutathione metabolism: glutathione peroxidase reduces hydrogen peroxide with the participation of glutathione, glutathione reductase restores the disulfide bond of oxidized glutathione, and glutathione transferase performs glutathione attack on cellular toxic compounds, along with detoxification and xenobiotic degradation [122]. Upon H_2_O_2_-induced OS, the transcriptional activity of glutathione reductase gene increased by more than six times in *L. plantarum*, and the transcription of glutathione peroxidase also increased [123]. Killisaar et al. were the first to report that *L. fermentum* ME-3 possesses a fully functional GSH system comprising both a GSH peroxidase and a GSH reductase [124]. Lactobacilli contain the genes for the synthesis of glutaredoxin—small redox enzymes oxidized by substrates, and reduced non-enzymatically by glutathione [125].

Most studied bifidobacteria do not produce any detectable amounts of GSH, but the gene for the protein with the prokaryotic glutathione synthetase domain, involved in the synthesis of glutathione, is located in the genome of the *B. dentium* JCVIHMP022 strain (BioCyc database). Most bifidobacteria contain the gene *gsiA* for glutathione import via the ATP-binding protein that is involved in the transport of glutathione from the growth medium into the cell.

The enzymes described above determine the resistance to OS in bacterial cells. They can be important biomarkers for the selection of bacteria with AO properties (Table 2).

### 4.4. Other Probiotic Metabolites and Cellular Components with Antioxidant Properties

Apart from the endogenous enzymatic and non-enzymatic AOs, bifidobacteria and lactobacilli can produce a number of metabolites and cellular components that can block free radical oxidation reactions.

Peptides derived from hydrolyzed food proteins have been shown to possess AO activities that can impart protection against the peroxidation of lipids and fatty acids [126]. It has been observed that peptic digestion of casein liberates small peptides with radical scavenging activity [127]. Increased AO activity of milk following fermentation with commonly used dairy starter cultures, including *L. jensenii* and *L. acidophilus*, has been observed [23,128].

Production of bioactive peptides with AO properties by bifidobacteria is rarely reported in the literature. One example is a study that demonstrated the possibility of antioxidant peptide production from bovine casein by *B. longum* [129]. The subtilisin-like serine protease cell envelope protease (CEP) that catalyzes the cleavage of peptide bonds could be used by *B. longum* KACC91563 for the production of bioactive peptides with AO properties [130].

The AO ability of probiotic bacteria can be considered to be a result of the ability to produce amino acids in high quantities. Bifidobacteria exhibit the ability to produce amino acids with AO properties, such as cysteine and methionine [131]. Some bifidobacteria possess the genes involved in the reverse transsulfuration pathway, which produces cysteine from methionine using homocysteine as an intermediate. There are genes such as *ahcY* that encode an *S*-adenosylhomocysteinase, and *luxS*, which encodes a S-ribosylhomocysteinase for the *S*-adenosyl methionine cycle [130,131].

Tryptophan metabolites elicit AO and anti-inflammatory immune responses partially via activation of the Nrf2-ARE pathway. These metabolites increase expression of target genes in the NF-E2-related factor 2 (NRF2)-mediated AO pathway (HMOX1, HS3ST2, TXNRD1, MGST1, ZNF643, EPS8, and TIPARP), and upregulate the AhR-inducible gene CYP1A1 [132]. *L. reuteri* and *L. johnsonii* convert tryptophan to indole-3-aldehyde, which exacerbates inflammatory disorders and affects the intestinal tract. Tryptophan acts as a metabolic substrate for the production of AhR ligands, such as indole-3-aldehyde and indole-3-lactic acid, by members of *Lactobacillus*, and these ligands inhibit colonization by *Candida albicans* and uncultivable segmented filamentous bacteria [133]. AO abilities have been identified for some tryptophan metabolites, such as *p*-hydroxyphenylacetate [134], indoleacrylic acid [132], and indolepropionic acid [88]. The enzymes tyramine dehydrogenase (*hpa* gene), indolelactate dehydratase (from gene cluster (fldAIBC)), and phenyllactate dehydratase (from gene cluster (fldAIBC)) could be used by bifidobacteria for the production of these metabolites [132].

Recent studies have revealed that histamine also plays a role in the AO potential of lactobacilli. Supernatants containing different concentrations of histamine produced by *L. reuteri* strains increased the activities of CAT and SOD, as well as the phagocytic activity of human leucocytes [135,136].

Several studies have linked improvements in gut health with the increase in gastrointestinal AO capacity following intake of AOs such as polyphenols and tocopherols [137]. Polyphenols such as lignans and flavonoids—both products of fermentation of plant components by bifidobacteria—possess an AO effect [138,139]. In the study of Braune and Blaut, the ability of *Bifidobacterium* strains (*n* = 25) to produce lignan and flavonoids aglycones from flaxseed and soybean extracts were examined [140]. Most of the *Bifidobacterium* strains increased the concentrations of secoisolariciresinol, daidzein, genistein, naringenin, eriodyctiol, luteolin, and apigenin. Moreover, *B. pseudocatenulatum* and *B. breve* strains showed high production of herbacetin, increased the kaempferol concentration, and produced quercetin and quercetagetin. *Bifidobacterium* strains converted the glycosides of a wide range of flavonoids into their aglycones, increasing the AO activity and improving their bioavailability [141].

Individual strains such as *L. acidophilus*, *L. buchneri*, *L. casei*, and *L. plantarum* are capable of carrying out O-deglycosylation of flavonoids. The strain *L. plantarum* IFPL935 was able to perform deglycosylation of C-glycosides of flavonoids. The enzymes β-glucosidases and α-rhamnosidases participate in this process. Some strains catalyze only single steps of known transformation pathways, while others catalyze complete conversion to typical degradation products [140]. The strain *L. pentosus* NGI01 produced high yields of hesperetin and quercetin from hesperidin and rutin, respectively [142].

Ferulic acid (FA) is a natural phenolic acid that is present in abundance in many types of foods—namely, cereals, fruits, and coffee. FA is a potent AO that can eliminate free radicals via neutralization reactions [143]. Some probiotic bacteria produce feruloyl esterase (FE), which hydrolyzes and releases FA from its bound state [144], thereby conferring health-beneficial AO properties. Based on qualitative precipitation and quantitative HPLC assays, *L. fermentum* NCIMB 5221 was found to produce the most active FE among several bacteria tested [145], and antioxidant capacity tests verified its significant AO activity. Oral administration of *L. fermentum* CRL1446 to mice increased total intestinal FE activity, decreased the basal percentage of plasma lipoperoxides, and increased glutathione reductase activity, thus improving oxidative status. Probiotic strains might secrete FE enzymes directly into the intestine, or via regulation of the intestinal microbiota stimulating FE activity [146].

Strains of *B. longum* use hydroxycinnamic acid esterase (gene *cae*A) to release hydroxycinnamates from plant-based dietary sources [141]. β-glucosidase is used by bifidobacteria to produce flavonoids and lignans from various plant sources [137,138,147]. Urolithin A (UroA) is a microbial metabolite derived from polyphenolics (e.g., ellagitannins/ellagic acid) of pomegranate and berries by dehydroxylase from urolithin C. UroA displays potent anti-inflammatory, antioxidative, and antiaging properties [148]. *B. pseudocatenulatum* INIA P815 can use up to 9 and 10 dehydroxylases to convert ellagic acid into UroA [149].

Some lactobacilli produce organic pigments called carotenoids, which are associated with AO activity. *Lactiplantibacillus pentosus* KCCP11226 harbors C30 carotenoid biosynthetic (*crt*M, *crt*N) genes, which are not common to most *Lactobacillus* species. This strain demonstrated the highest survival when exposed to OS, and the highest ability to scavenge DPPH free radicals. Carotenoid production in the strain was increased following exposure to 7 mM H_2_O_2_ [150].

Probiotic bacteria, as members of the gut microbiota, are capable of synthesizing vitamin K and most of the water-soluble B vitamins.

*B. bifidum*, *B. breve*, *B. adolescentis*, *B. infantis*, and *B. longum* produce the vitamins nicotinate, thiamine (B_1_), pyridoxine (B_6_), folate (B_9_), and cobalamin (B_12_) [147].

Folic acid (B_9_) increases the resistance of lipoproteins to oxidation [151]. The *pabC* gene encoding 4-amino-4-deoxychorismate lyase for folate production was found in the genomes of *B. adolescentis* ATCC15703 and *B. pseudocatenulatum* [152]. Wild-type lactobacilli cannot synthesize folate. *L. plantarum* constitutes an exception among lactobacilli, since it is capable of folate production in the presence of para-aminobenzoic acid [153].

Vitamin B_6_ plays an important role in the mechanism of AO activity [147]. The synthase (pyridoxal 5′-phosphate synthase PdxS subunit) (*pdxS* gene)5′-phosphate synthase PdxT subunit (*pdxT* gene) is involved in vitamin B_6_ production in bifidobacteria [152].

Cobalamin (vitamin B_12_) possesses AO properties [154]. The main producers of vitamin B_12_ are *B. animalis*, *B. infantis*, and *B. longum*. The enzymes cobaltochelatase and adenosylcobyric acidsynthase are used to synthesize adenosylcobalamin [155]. Lactobacilli are traditionally known to beauxotrophic for cobalamin. However, individual strains of *L. reuteri*, *L. fermentum*, *L. buchneri*, *Lentilactobacillus hilgardii*, and *L. brevis* were able to synthesize cobalamin [156]. Approximately 30 genes participated in the de novo synthesis of vitamin B_12_ [157].

Riboflavin participates in various redox reactions and energy utilization. The enzymatic activities required for the biosynthesis of riboflavin from guanosine triphosphate (GTP) and ribulose-5-phosphate are encoded by the genes *rib*G, *rib*B, *rib*A, *rib*H, and *rib*C. The ability to synthesize riboflavin has been shown in many lactobacilli: *L. plantarum*, *L. fermentum*, *Limosilactobacillus mucosae*, *L. acidophilus*, etc. The amount of synthesized riboflavin reached 2.36 mg/L or more, depending on the conditions of cultivation [158].

Vitamin K2 (menaquinone) is produced by bacteria in the intestine, and plays an important role in electron transport. There is one account of production of menaquinone by individual members of lactobacilli [159]. Production of vitamin K2 by *L. fermentum* LC272 reached 184.94 μg/L in Rogosa medium [160].

Some cell wall components of probiotic bacteria exhibit AO properties. Exopolysaccharides (EPSs) are a group of carbohydrate polymers that play important roles in biofilm formation and cell adhesion. EPSs that are typically released by probiotic bacteria potentially play a role in oxidative stress reduction [161,162,163]. The AO activity of EPSs of lactobacilli has been repeatedly shown. EPSs are synthesized by a wide range of lactobacilli, including *L. plantarum*, *L. helveticus*, *L. gasseri*, and *L. sakei* [164]. In vitro testing of the AO activity of EPS-1 of *L. helveticus* KLDS1.8701 demonstrated strong scavenging properties on 2,2-diphenyl-1-picrylhydrazyl radicals, superoxide radicals, and hydroxyl radicals, as well as chelation of ferrous ions. Mice injected with D-gal EPS-1 showed significantly attenuated indicators of excessive oxidation, such as decreased organic index, liver injury, and liver oxidative stress. EPS-1 supplementation shifted the gut microbiota composition to that of the control group [165]. The synthesis of EPS is encoded by many genes, which are mainly organized in operons [166,167].

Many organisms can fully compensate for the loss of enzymatic AO defenses by accumulating metabolites and Mn^2+^. There is mounting evidence that Mn–Pi (orthophosphate) complexes act as potent scavengers of superoxide in all three branches of life. Moreover, it is evident that Mn^2+^ complexes with carbonates, peptides, nucleosides, and organic acids can also form catalytic Mn-AOs, pointing to diverse metabolic routes to resistance to OS. Mn can serve as an O_2_ scavenger within SOD-deficient lactobacilli cells, such as *L. plantarum*. It has been demonstrated that the activity of MnSOD is dependent on the intracellular concentration of Mn^2+^ [168]. Both the fermented supernatant and the cell homogenate of *L. plantarum* MA2 strain lacking SOD exhibited superoxide dismutase activity [169]. In *L. plantarum*, which lacks AO enzymes, Mn-antioxidants can serve as additional protection when enzymatic antioxidants are insufficient [170]. In *L. plantarum*, which lacks AO enzymes, Mn-antioxidants can serve as additional protection when enzymatic antioxidants are insufficient [170]. Manganese transporters encoded by *mnt*H1-*mnt*H2, and ABC-type manganese transporters encoded by the *mts* CBA cluster of *L. casei* Shirota, are involved in the accumulation of intracellular manganese, and are necessary for aerobic growth of the strain [171]. Manganese can protect bifidobacteria from OS by acting as a scavenger of both O_2_ and H_2_O_2_, in addition to several crucial roles in biological systems. The expression of the *znt*A1 (BBMN68_1149) gene, encoding a homologue of the P-type ATPase that may be involved in taking up Mn^2+^, was upregulated 2.01-fold following exposure to oxygen in *B. longum* BBMN68 for 60 min [105]. In addition, *B. longum* BBMN68 grew faster in MRS broth supplemented with Mn2+ upon exposure to 3% oxygen [105].

### 4.5. Effects on Cellular Receptors and Regulation of Internal Signal Transduction Systems of Eukaryotic Cells

Probiotic bacteria can stimulate the activity of host AO enzymes. Increased activity of SOD, catalase, GSH S-transferase, GSH, and GSH peroxidase following *Lactobacillus* supplementation have been observed not only in blood serum, but also in diverse tissues—including the liver—in various animals [172].

**Figure 2:**
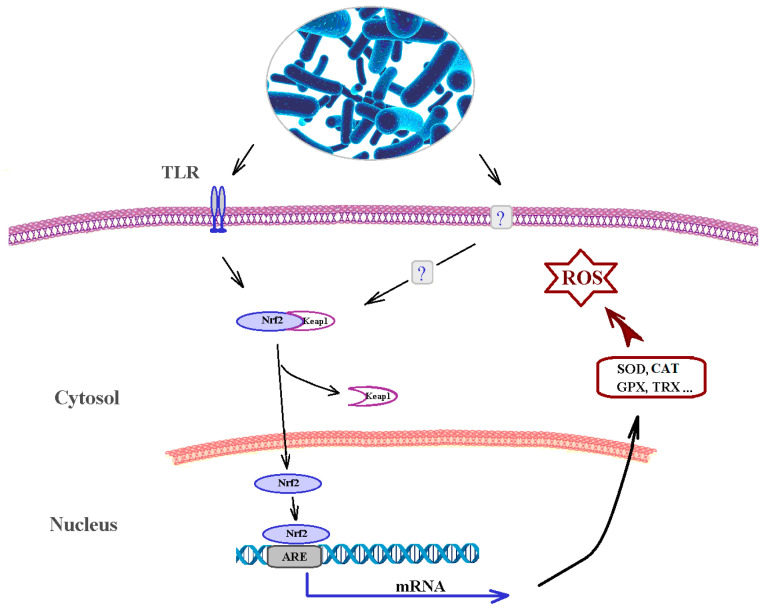
Effects of lactobacilli and bifidobacteria on cellular receptors and regulation of internal signal transduction systems of eukaryotic cells. ?: identified but undefined receptors and signaling molecules.

In recent years, both in vivo and in vitro studies have reported that probiotic bacteria can protect against OS through regulation of the Nrf2-Keap1-ARE pathway [30] (Figure 2). A number of signaling pathways associated with the AO mechanisms of probiotic bacteria in host cells also include silent information regulator 1 (SIRT1), mitogen-activated protein kinase (MAPK), and protein kinase C (PKC).

Nrf2-Keap1-ARE is an AO system that enables signal transmission from the outside into the cell and the nucleus. Under normal conditions, Keap1 is associated with Nrf2. Following ROS infiltration into the cell, the bond between Keap1 and Nrf2 is cleaved, and Nrf2 is transported into the nucleus, where it binds to ARE (antioxidant responsive element) sequences, activating the transcription of ARE-driven genes encoding AO enzymes [173]. Animals treated with *L. plantarum* CAI6 or *L. plantarum* SC4 demonstrated increased levels of Nrf2 in the liver and kidneys compared to the control group [29]. Saeedi et al. showed that oral administration of *L. rhamnosus* GG induced Nrf2 in the livers of normal mice, and this activation was sufficient to protect against two different models of acute oxidative liver damage (acetaminophen overdose and acute ethanol toxicity) [174].

SIRTs are an evolutionarily conserved family of NAD-dependent protein deacetylases that play an important AO role in mammalian and bacterial cells through the regulation of key genes and molecules that are an integral part of redox homeostasis [175]. Functional studies of probiotic SIRs are rare. However, there is a hypothesis that SIR2 plays the same AO role in probiotics as in eukaryotes. Guo et al. demonstrated that genes similar to SIR2 exist in *B. longum* and *L. acidophilus*, and are likely to increase aerotolerance by increasing AO enzyme activity [176]; these authors demonstrated that *B. longum* SIR2 upregulated the expression and activity of AO enzymes by deacetylating of the transcription protein SigH (σ^H^). Moreover, *B. longum* SIR2 can deacetylate the transcription factor FOXO3a in HEK293T cells, which mediates the gene expression of AO compounds. In vitro experiments on human T cells showed that SIR2 of *B. longum* can activate MnSOD/SOD2 and CAT, reducing the levels of ROS in human cells [176]. The administration of the probiotic formulation LAB51—consisting of bifidobacteria, lactobacilli, and *Streptococcus thermophilus*—to a mouse clearly reduced OS, which was mediated by increased activity and expression of SIRT1 [110]. The *sir2* gene encoding NAD-dependent protein deacetylase of the SIR2 family was found in the genome of *B. longum* LTBL16 [176,177]. SIR2 proteins can improve foxo-dependent transcription of antioxidants. *B. longum* with the *sir2* gene can eliminate free radicals from human cells, thus slowing down aging and reducing the incidence of cancer, heart disease, and other diseases [177]. Knockout of *sir2* from *L. acidophilus* decreases the AO activities of the deficient strain, while the reintroduction of LA-sir2 restores the strain’s AO activities [176].

Mitogen-activated protein kinases (MAPKs) are involved in many signaling pathways, including those associated with OS. MAPKs include four subfamilies, the best characterized of which are the extracellular regulated protein kinases (ERKs), c-jun N-terminal kinases (JNKs), and p38-MAPK; these can be activated by a variety of stimuli [178]. Tao et al. suggested that pretreatment of cells with *L. rhamnosus* GG-CM alone activated all three MAPKs that they studied [179]. Following administration of *L. gasseri* SBT2055 (LG2055) to *C. elegans*, SKN-1 (an Nrf ortholog) was activated, which induced the transcription of AO genes via p38, the MAPK pathway [180]. Another study discovered that LG2055 activated JNK, while inhibiting JNK led to the suppression of Nrf-2 ARE signaling activation and the protection against OS in mammalian cells [181].

PKC is a family of protein kinases that phosphorylate hydroxyl groups of serine and threonine residues in proteins. PKC is a target of redox modification for its unique structural features [182]. Zhou et al. showed that administration of *L. plantarum* improved intestinal barrier function and OS in a rat model of obstructive jaundice by strengthening the expression and activity of the PKC pathway [183]. Epithelial barrier disruption induced by H_2_O_2_ can be improved using the soluble proteins p40 and p75, produced by *L. rhamnosus* GG through a PKC- and MAPK-dependent mechanism [184].

NF-κB is the eukaryotic transcription factor that responds to OS. ROS can activate NF-κB entailing the expression of inflammatory cytokines. The probiotic formulation VSL#3—containing lactobacilli, bifidobacteria, and streptococci—is able to inhibit NF-κB and induce heat shock proteins in the epithelial cells of the colon [185].

The interaction of microbial products with Toll-like (TLR) receptors promotes the release of signaling molecules that lead to the activation of the Nrf2-Keap signaling pathway. Dissociation of the Nrf2-Keap complex can occur under the influence of various factors: phosphorylation of Nrf2 by various protein kinases (PKC: protein kinase C; JNK: a group of stress activated kinases and other factors; SOD: superoxide dismutase; CAT: catalase; GSH: reduced glutathione; GPX: glutathione peroxidase; TRX: thioredoxin; NRF2: nuclear factor erythroid 2-related factor 2; Keap1: Kelch-like ECH-associated protein 1,arepressor protein associated with Nrf2; ARE: antioxidant-responsive element (ARE-genes).

### 4.6. Impact on the Permeability of the Intestinal Barrier

The main function of the intestinal barrier is to protect internal organs from harmful agents. An increase in intestinal permeability leads to the infiltration of endotoxins and other microbial metabolites into the bloodstream, and translocation of microorganisms that, via stimulation of TLR, initiate cellular immune responses, activate macrophages, and provoke inflammation and OS [186]. A dysfunctional barrier, as observed in several diseases, is often the cause of an increase in OS, eventually leading to dysbacteriosis [187]. One of the major functions of probiotic bacteria is metabolism of dietary components, which leads to the generation of active metabolites that regulate barrier function [188].

Conjugated fatty acids and indole derivatives—both metabolites of intestinal lactobacilli and bifidobacteria—are involved in the regulation of intestinal permeability [188]. Conjugated fatty acids, such as conjugated linoleic acid (CLA), are known to influence gut barrier function. Treatment with the trans-10 CLA isomer caused a redistribution of ZO-1 and OCLN and increased paracellular permeability in Caco-2 colon epithelial cells [189]. A gene encoding for linoleic acid isomerase was found in the genomes of different species of bifidobacteria [190,191].

Small-molecule metabolites, produced by gut microbes derived from dietary tryptophan (indole-3-ethanol, indole-3-pyruvate, and indole-3-aldehyde), improve intestinal barrier integrity and protect against inflammation.

Metabolites of lactobacilli and bifidobacteria—such as secreted proteins, bacteriocins, and organic acids—increase mucus secretion by goblet cells’ production of antimicrobial peptides, as well as expression of tight junction proteins [192]. Extracellular proteins secreted by *L. plantarum* BMCM12 effectively keep pathogens from adhering to epithelial cells. The soluble protein HM0539, derived from *L. rhamnosus* GG, mediates tight junction expression and mucus secretion [193]. *B. infantis* secretes proteins that positively regulate occludin and ZO-1 proteins, and increase TER, thus reducing colonic permeability [194]. Bacterial-derived butyrate increased the expression of tight junction proteins in vivo [195], and stimulated goblet cells to secrete mucin—especially MUC2 [196]. Bifidobacteria produce acetate and lactate that can be converted into butyrate by other colonic bacteria via cross-feeding [41]. The surface components of probiotic cells—such as membrane proteins, capsule polysaccharides, flagella, and pili—form molecular patterns that specifically bind to pattern recognition receptors and regulate signaling pathways, increasing the levels of cytokines countering inflammation and enhancing the function of the gut epithelium. Microintegral membrane proteins of *L. plantarum* can restore injury caused to tight junctions by increasing the expression of JAM-1, occludins, and claudin-1 [197]. Tad pili of bifidobacteria can stimulate neonatal mucosal growth and intestinal maturation [198].

**Table 2:** A gene catalog comprising the key bacterial enzymes relevant to antioxidation.

Enzyme Name	Function	Gene	Strain	References
Superoxide dismutase	Superoxide anion scavenging.	*sod* LSEI_RS08890	*L. paracasei* ATCC 334	[168]
Catalase manganese-dependent	Catalyzes the decomposition of hydrogen peroxide to water and oxygen.	C1940_16840	*L. plantarum* LB1-2, Plasmid pLB1-2A	[46]
Catalase heme-dependent	Catalyzes the decomposition of hydrogen peroxide to water and oxygen.	*kat* Lpsk_RS08010	*L. plantarum* 90sk	[199]
Ferredoxin	Iron chelation activity.	BL1563	*B. longum* NCC2705	[83,84]
Peroxidase (thiol peroxidase)	Shows substrate specificity toward alkyl hydroperoxides over hydrogen peroxide.	*tpx*, Lb15f_RS10100	*L. brevis* 15f	[95]
Peroxidase (DyP-type haem peroxidase)	Wide substrate specificity, degrades the typical peroxidase substrates.	BWL06_08750	*L. plantarum* KLDS1.0391	[199]
Glutamate–cysteine ligase (γ-glutamylcysteine synthetase)	Glutathione synthesis, first stage.	*ghsA* AAX72_RS0316 HMPREF9003_RS10030	*L. brevis* 47f *B. dentium* JCVIHMP022	[119,199], BioCyc
gamma-glutamate-cysteine ligase/glutathione synthetase	Glutathione synthesis, both stages.	*ghsF*(AB), BWL06_02245,	*L. plantarum* KLDS1.0391	[119,120]
Glutathione peroxidase	Reduces glutathione to glutathione disulfide; reduces lipid hydroperoxides to alcohols.	*gpo*, BWL06_06975	*L. plantarum* KLDS1.0391	[122,199]
Glutathione S-transferase	Catalyzes the conjugation of the reduced form of glutathione (GSH) to xenobiotic substrates.	*gst*, LCA12A_RS05970	*L. casei* 12A	[122]
Glutathione reductase	Catalyzes the reduction of the oxidized form of glutathione (GSSG) to the reduced form.	*gshR/gor*, BWL06_06300, BWL06_09445	*L. plantarum* KLDS1.0391	[126,199]
Thiol reductant ABC exporter subunit CydC	Glutathione import.	*cydC*, C0965_RS00870	*L. fermentum* U-21	[121]
Thiol reductant ABC exporter subunit CydD	Glutathione import.	*cydD*, C0965_RS00865	*L. fermentum* U-21	[121]
Glutaredoxin	Reduce dehydroascorbate, peroxiredoxins, and methionine sulfoxide reductase. Reduced non-enzymatically by glutathione.	*grxA* ACT00_RS12315 *grx*1, *grx*C2 BBMN68_1397	*L. rhamnosus* 313 *B. longum* BBMN68	[83,105,126]
Glutaredoxin-like NrdH protein	Characterized by a glutaredoxin-like amino acid sequence and thioredoxin-like activity profile. Reduced by thioredoxin reductase.	*nrd* HC0965_RS00895 BL0668	*L. fermentum* U-21 *B. longum* NCC2705	[121]
Peroxiredoxin (alkyl hydroperoxide reductase subunit C)	Reduces H_2_O_2_, organic peroxides, and peroxynitrite.	*tpx* (*ahp*C), C0965_RS09890, *ahp*C, BL0615	*L. fermentum* U-21 *B. longum* NCC2705	[105,106,108]
Alkyl hydroperoxide reductase, subunits F	Catalyzes the NADH-dependent reduction of the peroxiredoxin AhpC.	*ahpF*, LM010_05765	*L. manihotivorans* LM010	[200]
Peroxideroxin OsmC family	Peroxidase activity with a strong preference for organic hydroperoxides.	C0965_RS08900 BLI010_09070	*L. fermentum* U-21 *B. infantis* JCM 7010	[200]
Peroxiredoxin Q/BCP	Protein reduces and detoxifies hydroperoxides, shows substrate selectivity toward fatty acid hydroperoxides.	LTBL16_ 000976 BL0615	*B. longum* LTBL16 *B. longum* NCC2705	[83,104,177]
Thioredoxin	Reduction of disulfide bonds of other proteins by cysteine thiol–disulfide exchange.	*trxA*, *B*, BWL06_01960, BWL06_03620, BWL06_06900, BWL06_08715, BBMN68_991, BLD_ 0988	*L. plantarum* KLDS1.0391 *B. longum* DJO10A	[83,103,105,112,199]
Thioredoxin reductase	Reduction of oxidized thioredoxins and glutaredoxin-like NrdH protein.	*trx*C, BWL06_10585, *trx*B, BBMN68_RS07015 EH079_RS10430 BL0649	*L. plantarum* KLDS1.0391 *B. longum* BBMN68 *B. longum* LTBL16 *B. longum* NCC2705	[103,104,105,108,177,199]
NAD(P)H oxidase	Source of cellular reactive oxygen species, transfers electrons from NADPH to oxygen molecule.	*nox*, BWL06_00410 BWL06_08660 LTBL16_ 001911	*L. plantarum* KLDS1.0391 *B. longum* LTBL16	[95,108,177,199]
NAD(P)H peroxidase.	Reduces H_2_O_2_ to water.	BWL06_10580, BWL06_10615, BWL06_12965	*L. plantarum* KLDS1.0391	[199]
NADH flavin oxidoreductase	Enzyme reduces free flavins by NADH. Is inducible by the hydrogen peroxide.	BWL06_01550 BWL06_07320	*L. plantarum* KLDS1.0391	[199]
Pyruvate oxidase	Catalyzes the oxidative decarboxylation of pyruvate in the presence of phosphate and oxygen, yielding acetyl phosphate, carbon dioxide, and hydrogen peroxide.	BWL06_03605 BWL06_08165 BWL06_10985 BWL06_10995	*L. plantarum* KLDS1.0391	[199]
Dihydroorotate dehydrogenase	Generates H_2_O_2_-forming NADH oxidase activity and indirect production of H_2_O_2_.	BWL06_03855 BWL06_09870 *pyr*K CNCMI_0917 *pyr*D CNCMI_0378	*L. plantarum* KLDS1.0391 *B.bifidum* CNCMI-4319	[114,199]
Oxygen-dependent coproporphyrinogen III oxidase	Involved in detoxifying molecular oxygen and/or H_2_O_2_.	Balat_0893	*B. lactis* DSM 10140	[100]
Possible Class I pyridine nucleotide-disulfide oxidoreductase (PNDR)	Enzyme is involved in the cellular oxidative stress response.	BL1626 Lp19_3298	*B. longum* NCC 2705 *L. plantarum* 19.1	[105,115]
P-type ATPase	Transport of manganese to the bacterial cell.	*mntP* BWL06_09205 *zntA1* BBMN68_1149	*L. plantarum* KLDS1.0391 *B. longum* BBMN68	[105,199]
Manganese ABC transporter ATP-binding protein	Transport of manganese to the bacterial cell.	BWL06_12065	*L. plantarum* KLDS1.0391	[199]
ABC transporter	Transport of manganese to the bacterial cell.	BWL06_12070	*L. plantarum* KLDS1.0391	[199]
Metal ABC transporter substrate-binding protein	Transport of manganese to the bacterial cell.	BWL06_12075	*L. plantarum* KLDS1.0391	[199]
Ferritin; ferroxidase; DNA starvation/stationary phase protection protein	Enzymes catalyzes the oxidation ofFe2+ ions by hydrogen peroxide, which prevents hydroxyl radical production by the Fenton reaction.	*dps*, LBP_RS12440 A1F92_RS15895 BL0618	*L. plantarum* P-8 *L. plantarum* CAUH2 plasmid pCAUH203 *B. longum* NCC2705	[108,113]
DsbA family oxidoreductase	Catalyzes intrachain disulfide bond formation as peptides emerge into the cell’s periplasm.	*dsbA*, LBHH_RS12125, A1F92_RS15940 MCC00353_12020	*L. helveticus* H10, *L. plantarum* CAUH2 pCAUH203, *B. longum* MCC00353	[113] BioCyc
Hydrogen peroxide resistance protein	Upregulated by both oxygen and hydrogen peroxide stress.	*hprA1*	*L. casei* strain Shirota.	[112]
Transcriptional regulator. Copper transporting ATPase	Metabolism/chelation of copper ions.	*copR*, JDM1_2697, *copB*, JDM1_2696	*L. plantarum* JDM1	[86]
Ribonucleotide reductase	DNA oxidative damage-protective protein.	*nrd*A, BL1752 LBP_cg2187	*B. longum* NCC2705 *L. plantarum* P-8	[105,109]
Nucleotide triphosphate pyrophosphohydrolases	DNA oxidative damage-protective proteins.	*mut*T1	*B. longum* BBMN68	[105]
Phytoene synthase Phytoene desaturase	Biosynthesis of carotenoids.	*crtM*, GMA16_RS13840, *crtN* GMA16_RS13835	*L. plantarum* KCCP11226	[151]
Histidine decarboxylase	Synthesis of histamine.	LAR_RS09695	*L. reuteri* JCM 1112	[152]
NAD-dependent protein deacetylase of SIR2 family	Involved in the response to oxidative stress. NAD + -dependent deacetylation of σH and transcription factor FOXO3a. Improve foxo-dependent transcription of antioxidant enzymes and reduce ROS levels in cells.	*sir*2, LP_RS01895, LTBL16_ 002010	*L. plantarum* WSFS1 *B. longum* LTBL16	[82,176]
Linoleic acid isomerase	Partcipates in linoleic acid metabolism. Conjugated linoleic acid metabolites exhibit the ability to protect cells from oxidative effects.	*lai* CNCMI4319_0491 SN35N_1476	*B. bifidum* CNCM I-4319 *L. plantarum* SN35N	[190]
Cyclopropane-fatty-acyl-phospholipid synthase	Catalyzes cyclopropane fatty acid (cell-surface component) biosynthesis.	BBMN68_1705 EC76_GL001195 EC76_GL002960	*B. longum* BBMN68 *L. plantarum* ATCC 14917	[105]
Feruloyl esterase	Hydrolyzes and releases ferulic acid from its bound state.	LA20079_RS01515	*L. acidophilus* DSM 20079	[145]
Riboflavin biosynthesis operon	Riboflavin biosynthesis.	*ribA*, *B*, *H*, *G*, Lpsk_RS01975, Lpsk_RS01960, Lpsk_RS01970, Lpsk_RS01965	*L. plantarum* 90sk	[158]
Cobalamin biosynthesis	Cobalamin biosynthesis.	At least 30 genes	*L. reuteri* JCM 112(T)	[155]
Hydroxycinnamic acid esterase	Release of hydroxycinnamates from plant-based dietary sources.	*cae*A	*B. longum*	[141]
S-adenosylhomocysteinase, S-ribosylhomocysteinase	Synthesizes cysteine from methionine using homocysteine as an intermediate.	*ahc*Y, *lux*S BLIJ_2075 FC12_GL001705	*B. infantis* ATCC 15697 *L. paracasei* subsp. *tolerans* DSM 20258	[131]
Subtilisin-like serine protease, cell envelope protease	Catalyzes the cleavage of peptide bonds.	*aprE*, *cep*	*B. longum* KACC91563	[130]
Tyramine dehydrogenase	p-Hydroxyphenylacetate production.	*hpa*	*Bifidobacterium* spp.	[134]
Indolelactate dehydratase	Indoleacrylic acid production.	gene cluster (*fld*AIBC)	*Bifidobacterium* spp.	[132]
Phenyllactate dehydratase	Indolepropionic acid production.	gene cluster (*fld*AIBC)	*Bifidobacterium* spp.	[132]
4-Amino-4-deoxychorismate lyase	Tetrahydrofolate production.	*pabC* LOSG293_010660	*B. adolescentis* ATCC15703, *B. pseudocatenulatum* *Schleiferilactobacillus oryzae* JCM 18671	[148,149,150,151]
PLP synthase: pyridoxal 5′-phosphate synthase PdxS subunit, pyridoxal 5′-phosphate synthase PdxT subunit	Pyridoxal phosphate production.	*pdxS*, *pdxT*	*B. longum*, *B. adolescentis*	[147,150]
Cobaltochelatase, adenosylcobyric acid synthase	Adenosylcobalamin synthesis.	*cob*Q LSA02_15070	*B. animalis*, *B. infantis*, *B. longum*, *L. sakei* NBRC 5893	[152,154]
9 and 10-Dehydroxylase	Conversion of ellagic acid into urolithin A.		*B. pseudocatenulatum* INIA P815	[149]

## 5. Perspectives for the Applications of the Antioxidant Properties of Probiotic Lactobacilli and Bifidobacteria

Inflammation and OS are common symptoms of chronic diseases: autoimmune, neurological, cardiac, and oncological. The development of chronic diseases is often accompanied by dysbiosis or dysfunction of the gut microbiome [201]. Probiotic bacteria of the families *Lactobacillaceae* and *Bifidobacteriaceae* are promising candidates for antioxidant drugs [202,203]. The development of drugs aimed at eliminating the gut microbiome inflammatory phenotype will be facilitated considerably if novel methodological and conceptual approaches are implemented in the search for unique strains of probiotic bacteria; these include comparative analysis of the genomes of lactobacilli and bifidobacteria, as well as metagenomes of the gut microbiome of healthy people and patients with chronic inflammatory diseases using omics technologies. The characterization of the gut microbiome in health and disease is more likely to become possible when biomarkers of a dysfunctional microbiome are better understood.

Today, research is underway to identify the genes accounting for the neuromodulatory and immunomodulatory properties of the gut microbiome. The neuromodulatory potential of the human gut microbiome has been studied since the emergence of the concept of the gut–brain axis. Potential biomarkers that account for the neuromodulatory potential of a gut microbiome have been identified [38,204,205]. The immunomodulatory potential of the human gut microbiome—and lactobacilli and bifidobacteria, in particular—is an interesting and novel topic for research [206].

However, research on the antioxidant properties of bacteria lacks systemization. This review systematically summarizes the accumulated knowledge on the antioxidant potential of bacteria, putting together a catalog of genes that encode proteins possessing antioxidant potential. The gene catalog could serve as a tool for the characterization of the antioxidant potential of the gut microbiome in health and disease.

The assessment of the antioxidant potential of the gut microbiome and probiotic bacteria is enabled by in silica analysis and development of algorithms. The first step is to identify the genes encoding products possessing antioxidant properties in the sequenced genomes of probiotic bacteria. The second step is to employ proteomic and metabolomic analyses to identify extracellular proteins and other compounds possessing antioxidant activity. The third step involves the assessment of the antioxidant properties of selected strains of probiotic bacteria in vitro, using cell lines and model organisms. This approach proved effective for the selection of strains such as *L. brevis* 47f and *L. fermentum* U-21, which possess outstanding antioxidant properties [61,71,72,206,207,208,209].

Correction of the gut microbiome of patients with chronic inflammatory diseases characterized by an imbalanced antioxidant system should be carried out using strains of probiotic bacteria with selected antioxidant properties. The gut microbiome of people resistant to OS can be mined for unique strains that can be used for the treatment of patients with chronic inflammatory diseases using a gut-microbiome-based approach.

The COVID-19 pandemic is a serious threat to public health, and not only because of the deaths numbering in the hundreds of thousands, but also because of the post-COVID-19 conditions that have complicated the lives of millions of people after they were infected. Complications of COVID-19 include autoimmune, cardiological, oncological, neurological, and chronic inflammatory conditions [210,211]. Public health systems around the world face a difficult task of rehabilitating hundreds of millions of people afflicted by COVID-19. The antioxidant properties of probiotics based on lactobacilli and bifidobacteria remain underestimated in this field [212]. For instance, the FN3 protein derived from *B. longum* GT15 has been shown to selectively bind to tumor necrosis factor alpha (TNF-α) [213,214]. New perspectives emerge for the use of components of lactobacilli and bifidobacteria, rather than using them as live cultures. These are known today as postbiotics, which are defined as metabolites and cell components conferring health benefits [215,216]. Postbiotics are a promising field of research for future pharmaceuticals and functional foods possessing antioxidant properties for the treatment of depressive disorders [217]. The potential of postbiotics can be harnessed by packing them into nanostructures, allowing them to be delivered to organs affected by inflammation [218]. The use of extracellular vesicles of Gram-positive probiotic bacteria, which can freely enter the bloodstream as well as tissues and organs of the human body, are another exciting area of research [219,220].

Metagenomics as a modern technique is extensively used not only to investigate differences in the microbiota composition in disease states compared to healthy individuals, but also to study functional genes of the gut microbiota. For this reason, it is desirable to use the metagenomic analysis of sequenced full-genomic bacterial DNA to study the AO potential of the gut microbiota. This approach can produce significant results when searching for target genes that are included in the reference gene catalog of the search tool. Table 2 provides a gene catalog comprising the key bacterial products relevant to the AO properties of probiotic lactobacilli and bifidobacteria. Orthologs of these genes could be identified in the available sequenced genomes of lactobacilli and bifidobacteria—representatives of the human intestinal microbiota—so as to search for them in the gut microbiota for the identification of the next potential AO biomarkers. An example of using a catalog of genes involved in the production of compounds related to ASD is presented in the study of Averina et al.; the use of such a methodological approach appeared to be effective for detecting significant changes in the metagenomic signature of the gut microbiota of children with ASD in comparison with neurotypical children [38]. Valles-Colomer et al. also used a catalog of bacterial genes encoding metabolites correlated with depressive disorders, in order to search for associations between the gut microbiota and depression [221]. Knowledge about AO bacterial markers can be used to diagnose OS, and also to provide indicators to monitor and guide individual therapy in the clinic.

## 6. Conclusions

Multiple in vivo and in vitro studies have demonstrated that lactobacilli and bifidobacteria, along with their components, possess outstanding antioxidant capacity that provides a certain degree of protection of both their own cells and those of their hosts against oxidative damage. An extensive body of research proves that probiotic bacteria are capable of imparting AO benefits to the human organism, preventing diseases associated with OS. Genomic, transcriptomic, and proteomic analyses of probiotic strains of lactobacilli and bifidobacteria allowed the detection of various intrinsic defense systems that protect cells from OS. The AO activity is more pronounced in lactobacilli, which is related to the fact that they are facultative anaerobes or microaerophiles. AO enzymes such as thioredoxin and GSH-glutaredoxin systems—and to a lesser extent superoxide dismutase and catalase—determine the AO properties of lactobacilli. The AO enzymes alkyl hydroperoxide reductase, thioredoxin reductase, and NADH oxidase are more common in bifidobacteria. The principal known mechanisms of AO activity employed by probiotic bacteria to reduce OS in the human organism include the regulation of complex signaling networks—mainly redox signaling of Nrf2—increase in AO enzyme levels, scavenging of ROS via different pathways, chelation of metal ions, improvement of gut permeability, and modulation of the intestinal microbiota. Cell wall components and metabolites of lactobacilli and bifidobacteria (e.g., EPS, tryptophan metabolites, histamine) contribute to an increase in the AO activity of host cells by acting on cellular receptors and regulating internal signal transduction. However, the AO action of probiotic bacteria in the human body has not been fully elucidated. In the near future, it is necessary to carry out comprehensive studies of the ways in which bacteria confer protection from OS to both their own cells and those of their hosts.

The studies of the antioxidant properties of bacteria, which allow for the identification of biomarkers of the antioxidant potential of lacto- and bifidobacteria strains, as well as the human gut microbiome, have not yet been systematized. In this review, the accumulated knowledge on the antioxidant potential of bacteria is systematized and a catalog of genes for antioxidant products of bacteria is presented, which can be used to characterize the antioxidant potential of the microbiome, including lactobacilli and bifidobacteria. The purpose of this review was to identify the AO biomarkers that characterize the potential of both individual strains and consortia of bacteria inhabiting the gut microbiota. These biomarkers can reflect the AO status of the host organism, as the analysis of metagenomic data from patients with different diseases was correlated with OS and altered gut microbiota. The creation of gene catalogs containing the antioxidative genes will allow for the full deciphering of metagenomic data. Mean while, defining the metagenomic AO signatures of lactobacilli and bifidobacteria in the norm is crucial for singling out the genes with diagnostic potential in the context of different diseases. The COVID-19 pandemic has mobilized the scientific community, business, and government agencies around the world to develop vaccines and drugs that can stop SARS-CoV-2 and reduce its socioeconomic consequences. Despite the progress in vaccine development, the number of new cases continues to rise. The data on the high risk of post-COVID-19 conditions after recovering from the disease are very alarming. Thus, intensive research is underway to reveal the link between inflammation, chronic diseases, and the gut microbiome.

It has been reported that following infection with SARS-CoV-2, the composition of the gut microbiome is altered, and characterized as dysbiotic. Overall, this is often accompanied by a decrease in the number of certain species of lactobacilli and bifidobacteria. Post-COVID-19 persistent dysbiosis can be a part of a multisystem inflammatory syndrome. It is well known that the gut microbiome is able to synthesize a complex of compounds with neuromodulatory, immunomodulatory, and antioxidant activity, which could characterize the gut microbiome.

Today, the gut microbiome is considered a valuable source for the development of pharmaceuticals, veterinary medicine, and functional foods. This is especially important in light of the urgent need for treating and rehabilitating a vast portion of the population in the post-COVID-19 era. Global advances in the study of the human microbiome, and the transition from classic probiotics such as dietary supplements to pharmabiotics, safe and with an established mechanism of action, open new horizons in personalized medicine.

## Data Availability

Not applicable.
